# Book Reviews

**Published:** 1915-10

**Authors:** 


### BOOK REVIEWS

DISEASES OF THE NOSE AND THROAT. By Algernon Coolidge, M. D., Professor of Larynology in the Harvard Medical School. 12mo. of 260 pages, illustrated. Philadelphia and London: W. B. Saunders Company, 1915. Cloth, $1.50 net.

This is a handy volume intended to serve as a reminder of things to the well-posted and leader into the great field of rhino-laryngology to the beginner. It treats of the subjects comprehensively and sets before the reader the settled opinions and assured facts of the times without going into the history of the speculations and the search that has established them.

Where there is still divergence of opinion as in the matter of tonsilectomy for example, it sets forth both sides and thus clears the way for individual choice in each case.

There is one error it perpetuates and that is a mention of the use of a spray of cocaine in any strength. We would not put forward a personal preference in the consideration of a book, but the use of cocaine is attended with such grave results that it is worth while even in a review to say that a spray of cocaine in any strength is dangerous.

For the specialist, the book presents briefly and concisely just the things his lay patients want to know about from time to time and it forms a ready and reliable reference should he desire to inform them from a book. For the general practioner it brings the general subject up-to-date and for the undergraduate it shows the way.

It is well-printed on paper without glare.

---

THE PRINCIPLES OF PATHOLOGIC HISTOLOGY, by F. B. Mallory, M. D., Associate Professor of Pathology, Harvard Medical School, Pathologist to The Boston City Hospital. Octavo Volume of 667 pages, 497 figures containing 683 illustration, 124 in colors and all but two original, printed directly in the text. Philadelphia and London: W. B. Saunders Company. Cloth, $5.00.

The author has planned the construction of this book with the system of studying the tissues in their earliest stages of development and watching their departure from the normal until their arrival at definite diseased states. . Thus, as he says in his preface, one can know all the conditions of a pathologic picture and become able to reason both ways from almost any manifestation it may make. The causes of disease may be checked before the disease, itself, appears and later on, one may be able to discover what has led to the disease to be treated and know how to handle it. The value of all this lies in the advice and counsel the pathologist and laboratory man may give to the clinician seeking information as to his patient. Moreover, a careful study of the book will enable the clinician to comprehend the more readily what the pathologist has to say and it will teach him when to seek the aid of the laboratory with the best results.

